# Thermographic Investigation of Elastocaloric Behavior in Ni-Ti Sheet Elements Under Cyclic Bending

**DOI:** 10.3390/ma18153546

**Published:** 2025-07-29

**Authors:** Saeed Danaee Barforooshi, Gianmarco Bizzarri, Girolamo Costanza, Stefano Paoloni, Ilaria Porroni, Maria Elisa Tata

**Affiliations:** Industrial Engineering Department, University of Rome “Tor Vergata”, 00133 Rome, Italy; saeed.danaeebarforooshi@alumni.uniroma2.eu (S.D.B.); gianmarco.bizzarri@alumni.uniroma2.eu (G.B.); stefano.paoloni@uniroma2.it (S.P.); ilaria.porroni@alumni.uniroma2.eu (I.P.); elisa.tata@uniroma2.it (M.E.T.)

**Keywords:** shape memory alloys, elastocaloric effect, green cooling, superelasticity, Ni-Ti

## Abstract

Growing environmental concerns have driven increased interest in solid-state thermal technologies based on the elastocaloric properties of shape memory alloys (SMA). This work examines the elastocaloric effect (eCE) in Ni-Ti SMA sheets subjected to cyclic bending, providing quantitative thermal characterization of their behavior under controlled loading conditions. The experimental investigation employed passive thermography to analyze the thermal response of Ni-Ti sheets under two deflection configurations at 1800 rpm loading. Testing revealed consistent adiabatic temperature variations (Δ*T_ad_*) of 4.14 °C and 4.26 °C for the respective deflections during heating cycles, while cooling phases demonstrated efficient thermal homogenization with temperature gradients decreasing from 4.13 °C to 0.13 °C and 4.43 °C to 0.68 °C over 60 s. These findings provide systematic thermal documentation of elastocaloric behavior in bending-loaded Ni-Ti sheet elements and quantitative data on the relationship between mechanical loading parameters and thermal gradients, enhancing the experimental understanding of elastocaloric phenomena in this configuration.

## 1. Introduction

The development of efficient and compact thermal technologies is a significant challenge of modern technology, especially for applications that require precise temperature control in confined spaces [1]. Traditional vapor-compression refrigeration systems rely on refrigerants with significant global warming potential and involve components that limit miniaturization possibilities. In light of the increasing environmental concerns and demand for compact thermal management solutions, researchers have made significant efforts to develop alternative thermal technologies that utilize solid-state refrigerants [2].

Shape memory alloys (SMA) are materials of interest for solid-state thermal applications because of their unique property known as elastocaloric effect (eCE), that arises from reversible stress-induced martensitic transformation [3]. This unique property of SMA, combined with their superelasticity, has made them valuable for various engineering applications, including self-centering seismic devices [4] and thermal management solutions that eliminate the need for traditional refrigerants [5].

Among various alloys exhibiting elastocaloric properties, Ni-Ti-based alloys are particularly attractive for thermal applications due to their significant achievable adiabatic temperature variations (up to 20 °C) under appropriate loading conditions [6] and their excellent mechanical properties [7].

The elastocaloric effect shown by these materials results from reversible stress-induced martensitic transformation. During mechanical loading of the alloy, an exothermic phase change occurs from austenite to martensite. When the load is removed, an endothermic reverse transformation takes place, producing a cooling effect [8].

The elastocaloric thermal cycle typically follows a Brayton thermodynamic cycle, including adiabatic loading (resulting in temperature increase), isobaric heat release, adiabatic unloading (producing temperature decrease), and isobaric heat absorption [9].

The influence of loading configuration on the thermomechanical behavior of SMA is particularly significant for elastocaloric applications. Most of the research so far has focused on uniaxial tensile or compressive loading configurations, approaches that present limitations in practical applications. As an example, conventional uniaxial tension loading, while producing substantial temperature variations, typically suffers from reduced fatigue life compared to compression or bending modes [10]. Compressive loading offers improved fatigue resistance but faces practical limitations related to buckling phenomena, especially in thin samples [11].

Some research groups have investigated elastocaloric behavior under different loading mechanisms. A pioneering study by Sharar et al. [12] examined a system utilizing Ni-Ti wire under bending loading modes, observing temperature variations of comparable magnitude but with significantly reduced actuation forces in the bending configuration. More recently, Cheng et al. [13] developed an analytical model introducing the concept of continuous rotating bending cantilever actuation system using Ni-Ti sheets, demonstrating both theoretical and experimental aspects of this approach. Li et al. [14] presented an innovative coil-bending air cooler with thermal responses under bending conditions.

While previous studies have demonstrated the feasibility of bending-mode actuation and explored continuous rotating configurations, challenges remain in obtaining detailed thermal characterization during cyclic loading. This research addresses these gaps through comprehensive spatiotemporal thermographic analysis, providing distinctive contributions in three key areas: detailed characterization of thermal gradient evolution and homogenization patterns, quantitative analysis of the relationship between deflection parameters and thermal response characteristics, and systematic evaluation of thermal homogenization dynamics during cooling phases. Unlike prior investigations that primarily focused on system-level performance metrics or proof-of-concept demonstrations, our study provides in-depth thermal mapping across the entire Ni-Ti sheet surface, revealing localized transformation patterns and thermal transport mechanisms.

The primary objectives include quantifying temperature variations achieved through cyclic bending loading, analyzing thermal homogenization dynamics during cooling cycles, assessing reproducibility and consistency of the elastocaloric response, and evaluating thermodynamic performance through systematic thermal characterization methodology.

This comprehensive spatiotemporal thermographic approach provides detailed experimental documentation of elastocaloric behavior in bending configurations, contributing quantitative thermal characterization data for Ni-Ti sheet elements under cyclic loading conditions. The methodology developed in this investigation offers a foundation for systematic assessment of thermal response characteristics in elastocaloric materials subjected to non-uniaxial loading scenarios.

## 2. Materials and Methods

In this section, the experimental methodology employed to investigate the elastocaloric effect in Ni-Ti shape memory alloy sheets subject to cyclic bending loading is presented. In detail, the Ni-Ti sheet, positioned in a cantilever configuration, is cyclically subjected to a bending stress generated by the rotation of the shaft of a laboratory’s lathe, while the infrared camera acquires the sheet’s surface temperature in real time. The experimental framework covers material preparation, specialized setup development for cyclic bending tests, and thermal imaging protocols designed to quantify the elastocaloric response under varying bending loading conditions.

### 2.1. Material Characteristics and Sample Preparation

The experimental investigation was conducted on commercial superelastic Ni-Ti sheets with dimensions of 100mm×20mm×1mm.

The material was specified as a standard Ni-Ti alloy optimized for superelastic applications, although precise chemical composition details were not disclosed by the manufacturer. The manufacturer’s specifications indicate characteristic transformation temperatures with martensite phase present below −10 °C and an austenite finish temperature ($A_{f}$) of 5 °C. This temperature profile ensures that, at ambient temperature and in the absence of an applied load, the material remains in the austenitic phase, providing optimal conditions for superelastic behavior and elastocaloric response.

**Figure 1:**
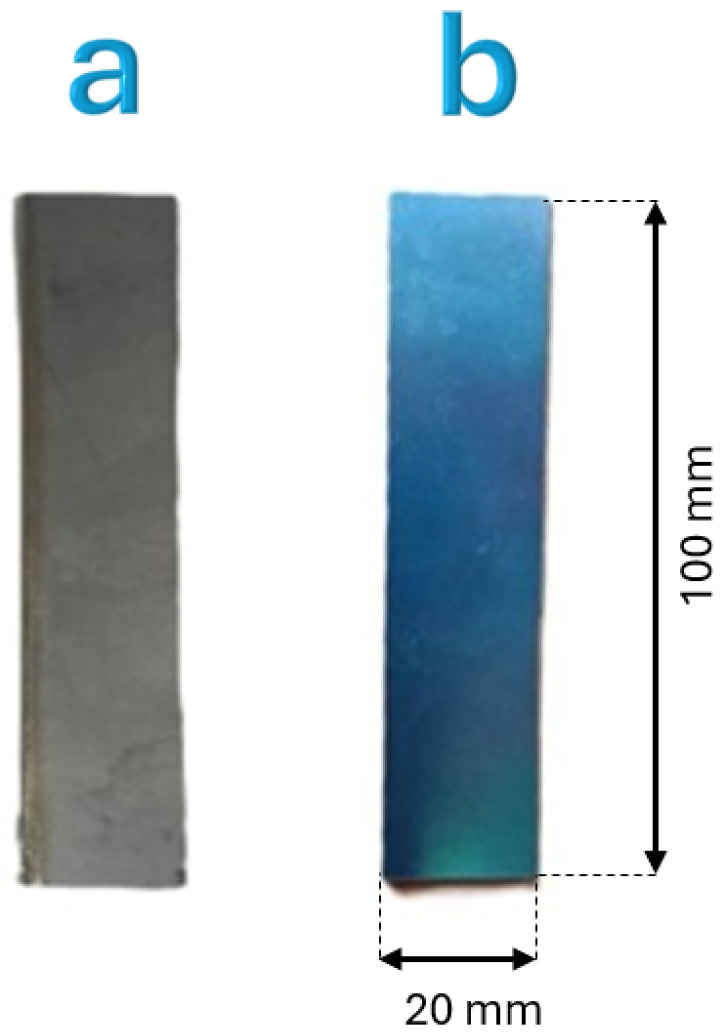
Ni-Ti sheet before (**a**) and after (**b**) the shape-setting heat treatment, showing characteristic surface appearance resulting from the heat treatment. Image previously published in [15].

The Ni-Ti sheets employed in this investigation are the same material used in a previous study focused on mechanical characterization under uniaxial tensile loading [15]. Figure 1 illustrates the appearance of the specimens before and after the shape-setting heat treatment at 500 °C. For a more detailed description of the material properties, including X-ray diffraction (XRD) analysis confirming the austenitic B2 phase, the reader is referred to [15].

Initial material characterization was performed to establish baseline properties prior to experimental testing. Vickers microhardness measurements, conducted according to UNI EN ISO 6507-1 standard [16], employed three distinct load levels (300 gf, 500 gf, and 1000 gf) with a standardized application time of 10 s and an indentation speed of 0.2 mm/min during loading and unloading phases. For statistical reliability, three series of indentations were executed at each load level, with diagonal measurements independently assessed twice to minimize measurement uncertainty. The as-received material exhibited an average hardness of 368.1 HV with a standard deviation of 12.6 HV, indicating suitable mechanical properties for elastocaloric applications.

For thermographic analysis, a thin graphite coating was applied to the sample surface, enabling accurate temperature measurements during the tests.

**Figure 2:**
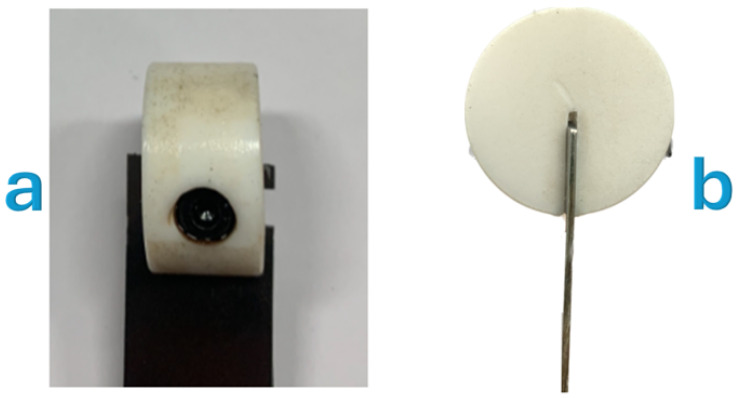
Views of the Derlin support with the Ni-Ti sheet and the inserted M6 threaded grain. (**a**) Side view. (**b**) Front view.

For the cyclic bending experiments, specimens were configured as cantilevers with a 52 mm free length, fixed at one end. To optimize the interface contact between the specimen and the loading system, a specially designed notch was carefully machined at the free end of the Ni-Ti sheet. This modification was crucial for minimizing frictional effects that could potentially serve as a primary heat source and interfere with elastocaloric measurements. The notch geometry facilitated the insertion of an M6 threaded pin, creating a robust mechanical coupling with a polyoxymethylene (POM, commercially known as Derlin) support component that interfaced with the eccentric cam in the experimental setup. This configuration, shown in Figure 2, ensured that the thermal response observed during testing could be predominantly attributed to the elastocaloric effect rather than frictional heating at the contact interface.

### 2.2. Experimental Setup for Cyclic Bending

**Figure 3:**
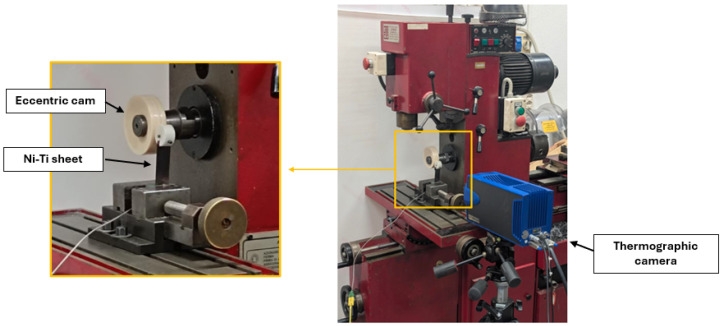
Experimental configuration showing the relative positioning of key components: eccentric cam mechanism for generating cyclic bending, Ni-Ti sheet in cantilever configuration, and infrared thermographic camera.

A specialized experimental apparatus was developed to investigate the elastocaloric effect in Ni-Ti sheets through cyclic bending. The setup utilized a modified commercial lathe as the primary mechanical actuation system, adapted to generate precise cyclic bending deformation of the Ni-Ti sheets, as shown in Figure 3.

The mechanical loading system comprised a rotating shaft connected to the lathe’s main spindle, equipped with an eccentric cam that transformed the rotational motion into cyclic bending deformation of the cantilever-mounted Ni-Ti sheet. The eccentric cam, with an eccentricity parameter (*e*) of 13.5 mm, was manufactured from polyvinyl chloride (PVC) and had a diameter of 24 mm and thickness of 28 mm. This configuration allowed the generation of controlled deflections at the free end of the specimen.

The specimen support system was carefully designed to minimize unwanted frictional heating that could interfere with the elastocaloric temperature measurements. After evaluating multiple materials, polyoxymethylene was selected for the interface component at the free end of the specimen due to its favorable combination of mechanical properties and low thermal conductivity. Material selection was guided by a detailed analysis of density, thermal conductivity, and mechanical stability parameters, with Derlin (density: $1.41\times 10^{-3}$ g/mm^3^) providing a 36.6% mass reduction compared to polytetrafluoroethylene (PTFE) alternatives while maintaining sufficient structural integrity.

The contact interface between the specimen and the eccentric cam underwent several iterations to achieve optimal mechanical coupling and minimize parasitic heating. The final design incorporated a specially machined notch at the free end of the Ni-Ti specimen, allowing secure connection to a Derlin support component via an M6 threaded pin. This configuration ensured effective force transmission from the rotating eccentric cam while significantly reducing frictional effects that could otherwise introduce measurement artifacts. Preliminary tests confirmed that this optimized interface effectively isolated the elastocaloric response from frictional heating influences.

### 2.3. Thermographic Measurement System

The elastocaloric effect was studied using passive infrared thermography. A CEDIP Jade II infrared camera was utilized for this research study, equipped with a Indium Antimonide (InSb) cooled detector sensitive in the mid-wavelength infrared spectrum (3.5–5.0 μm). This non-contact measurement approach enabled detailed thermal mapping across the entire Ni-Ti sheet surface without interfering with the material’s thermal response.

The thermographic acquisition system, shown in Figure 3, comprised several integrated components: the thermal imaging camera head with its cooled InSb sensor, a 24 VDC power supply, a dedicated signal processing unit, and a data acquisition computer running CIRRUS WIN ALTAIR software v5.50.013b for real-time image capture and analysis. The thermal images were digitized using a PG 9800 frame grabber (Cisco Systems, San Jose, CA, USA), providing a continuous stream of calibrated temperature data throughout the testing period.

Temperature calibration was performed using the CF Manager module within the ALTAIR software. The calibration procedure established the correlation between digital levels (DL) measured by the sensor and actual temperatures, accounting for object emission, ambient reflection, and atmospheric contribution. The camera’s integration time was standardized at 1200 μs for all measurements.

Data acquisition was conducted at a sampling frequency of 25 Hz (25 frames per second), resulting in 1500 images per minute. This sampling rate represented an optimal compromise between temporal resolution and data management considerations, allowing detailed characterization of the elastocaloric thermal evolution while maintaining reasonable file sizes. Specific acquisition time parameters were set as detailed in Table 1.

### 2.4. Operational Parameters and Testing Procedure

The analysis was conducted using two different preload configurations to investigate the effect of deflection on the elastocaloric response:

- Configuration 1: Total deflection (δ) of 31 mm.
- Configuration 2: Total deflection (δ) of 33 mm.

Table 2 summarizes operational parameters for the experimental setup. In both configurations, Ni-Ti sheets with an active free length of 52 mm were cyclical loaded by the eccentric cam at a rotational speed of 1800 rpm, corresponding to a loading frequency of 30 Hz.

The experimental setup, illustrated in Figure 3, shows the spatial arrangement of all key components.

**Figure 4:**
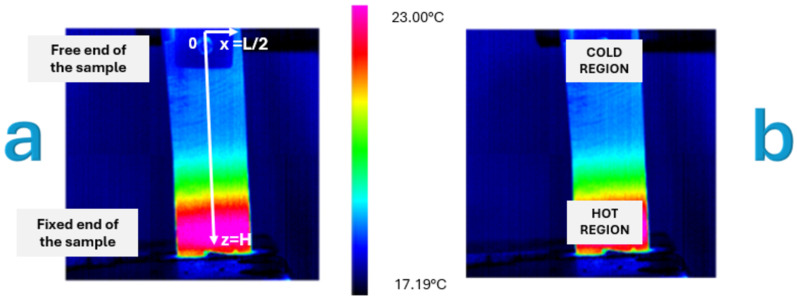
Thermographic visualizations of the Ni-Ti sheet showing the definition of coordinate system and regions of interest. (**a**) Thermal distribution with coordinate system showing z = 0 at the free end of the sheet and z = H at the fixed end. (**b**) Defined “hot region” at the fixed end and “cold region” at the free end.

A reference coordinate system, illustrated in Figure 4a, was established with the origin (z = 0) at the free end of the cantilever Ni-Ti sheet and the z-axis aligned with the sheet’s longitudinal direction, extending to z = H (where H = 52 mm) at the fixed base of the sheet. The x-axis was defined in the direction along the width of the Ni-Ti sheet, with x = 0 at the center and extending to x = ±L/2 (where L = 20 mm) at the lateral edges of the sheet.

For each configuration, the experimental investigation was structured into two distinct test types:

- Heating tests: two successive trials (30 s each) to evaluate the temperature increase due to the elastocaloric effect and assess measurement repeatability.
- Cooling tests: one extended trial (60 s each) to analyze the thermal homogenization dynamics after cessation of mechanical loading.

The analysis methodology for each test incorporated examination of three longitudinal thermal profiles:

- Central profile (x = 0).
- Two lateral profiles (x = ±L/2, with L = 20 mm).

Additionally, two regions of interest with equivalent areas (182.60 mm^2^) were defined to quantify the energy associated with the superelastic behavior. The selection of these regions was based on classical beam theory and preliminary thermal observations. The “hot region” was positioned at the fixed end (z = H) where maximum bending stress occurs, while the “cold region” was located at the free end (z = 0) where minimal stress is expected. The equivalent area of 182.60 mm^2^ was determined to ensure statistical significance of temperature measurements while maintaining adequate spatial resolution for capturing localized thermal effects.

Figure 4b displays the location of these regions on the Ni-Ti sheet, illustrating the thermal gradient development.

The actuation system enabled precise control of the loading parameters, with the capability to adjust both the deflection magnitude and the rotational speed, ensuring accurate and reproducible mechanical loading conditions essential for quantitative elastocaloric effect characterization.

### 2.5. Data Analysis Methodology

A standardized analysis sequence was employed for both heating and cooling tests. Temperature distributions were examined at key time points: t = 0 s and t = 30 s for heating tests, and t = 0 s and t = 60 s for cooling tests. Thermal evolution was characterized through three longitudinal profiles along the specimen (x = −L/2, x = 0, x = L/2) to assess the spatial distribution of thermal effects in the transverse direction.

The analysis bases on parameters calculated to quantify the thermal response. These include the following:

- Minimum, maximum, and mean temperatures along each profile;
- Standard deviations characterizing thermal homogeneity;
- Adiabatic temperature change ($\Delta T_{ad}$), defined as the difference between hot and cold region temperatures.

To ensure accurate comparison between different test configurations and repetitions, a set of dimensionless parameters was introduced:

(1) $$\gamma_{std}=\frac{\sigma_{hot}}{\sigma_{cold}}$$

(2) $$\gamma_{T}=\frac{{\bar{T}}_{hot}}{{\bar{T}}_{cold}}$$

(3) $$\Delta T_{\%}=\frac{|T_{1}-T_{2}|}{T_{1}}\times 100\%$$

where $\gamma_{std}$ represents the ratio between standard deviations in hot and cold regions, $\gamma_{T}$ denotes the ratio between mean temperatures, and $\Delta T_{\%}$ quantifies the percentage difference between temperatures in successive tests.

The reproducibility of the elastocaloric response was evaluated by calculating the mean value and standard deviation of $\Delta T_{ad}$ across repeated tests, with relative deviation expressed as a percentage of the mean:

(4) $$\Delta_{rel}=\frac{\sigma_{\Delta T_{ad}}}{\bar{\Delta T_{ad}}}\times 100\%$$

For cooling tests, particular attention was given to the thermal homogenization dynamics by monitoring the evolution of temperature gradients over time. The efficiency of the thermal homogenization process was quantified by comparing initial and final temperature differences between hot and cold regions.

The thermodynamic performance of the elastocaloric effect was assessed by calculating the heat generated during the martensitic transformation [10]:

(5) $$Q=\rho_{SMA}\cdot V_{SMA}\cdot c_{SMA}\cdot \Delta T_{ad}$$

where $\rho_{SMA}$ represents the experimentally determined density of the Ni-Ti alloy (6.45 g/cm^3^); $V_{SMA}$ is the volume of the material undergoing transformation; $c_{SMA}$ is the specific heat capacity (0.46 J/g·K, calculated as detailed in Appendix A using the methodology of Smith et al. [17]); and $\Delta T_{ad}$ is the measured adiabatic temperature change. These material properties are consistent with values reported in literature for superelastic Ni-Ti alloys [10].

It should be noted that the term “adiabatic temperature change” ($\Delta T_{ad}$) used throughout this work refers to quasi-adiabatic conditions, consistent with established terminology in elastocaloric literature. The rapid loading rate (30 Hz) ensures that the characteristic loading time is significantly shorter than the thermal diffusion time scale, minimizing heat exchange with the environment during the measurement period. This approach has been validated in previous studies and represents the standard methodology for characterizing elastocaloric effects in shape memory alloys [18].

## 3. Results

This section presents the obtained experimental results from both heating and cooling cycles, with particular focus on the adiabatic temperature variations ($\Delta T_{ad}$) observed under different deflection configurations. The thermogram reveals distinctive thermal patterns during cyclic loading, providing quantitative insights into the material’s temperature response, thermal homogenization dynamics, and overall thermodynamic behavior. Statistical evaluation confirms the consistency and reproducibility of the elastocaloric response across multiple tests, providing a foundation for subsequent analysis of the thermomechanical characteristics under cyclic bending conditions.

### 3.1. Heating Cycle Analysis

The analysis of heating cycles revealed significant elastocaloric effects in the Ni-Ti sheets subjected to cyclic bending loads. For each deflection configuration, two independent tests were performed to assess repeatability, with Tests 1 and 2 conducted at δ = 31 mm, Tests 3 and Test 4 performed at δ = 33 mm.

Table 3 summarizes the adiabatic temperature variations ($\Delta T_{ad}$) obtained from all four heating tests, showing both individual test results and averaged values for each configuration.

The following subsections present detailed temperature evolution data for each test configuration.

#### 3.1.1. Configuration 1 (δ = 31 mm): Test 1 and Test 2

**Figure 5:**
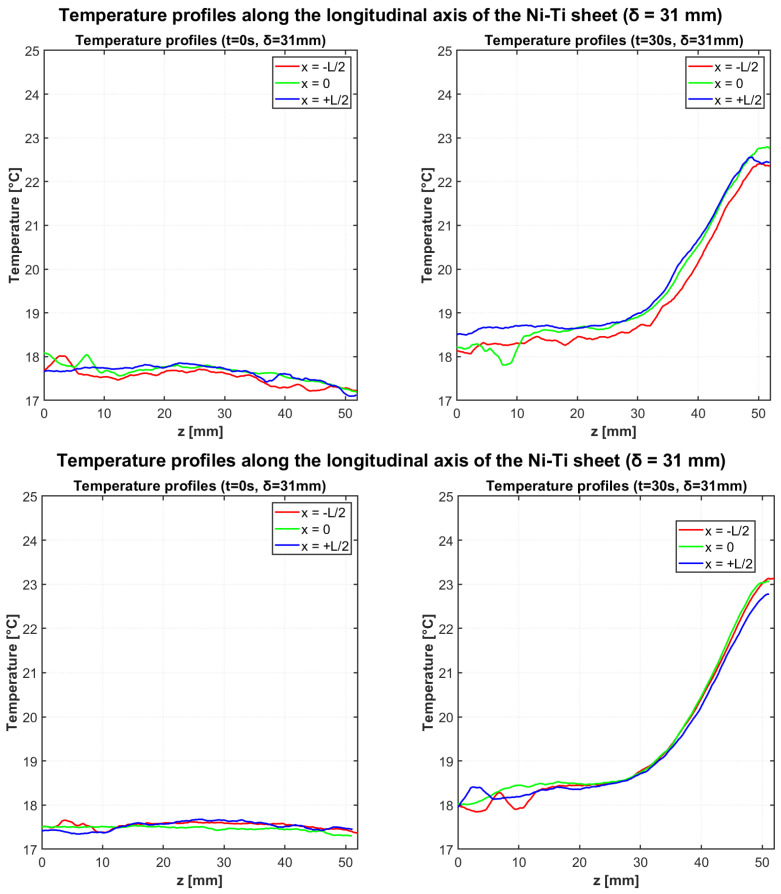
Temperature profiles along the longitudinal axis of the Ni-Ti sheet at t = 0 s (**left**) and t = 30 s (**right**) for two independent heating tests with deflection δ = 31 mm.

Figure 5 illustrates the temperature evolution along the longitudinal profiles for both independent heating tests with δ = 31 mm deflection. The three colored lines in all graphs represent measurements along different longitudinal profiles (x = −L/2, x = 0, x = L/2). All profiles demonstrate the development of thermal gradients during cyclic bending, with maximum temperatures occurring near the fixed end of the Ni-Ti cantilever beam.

Table 4 and Table 5 provide the quantitative characterization of the temperature profiles for both tests with δ = 31 mm configuration at the initial and final time points, respectively.

For Test 1, analysis of the specific regions reveals that the “cold region” temperature increased from 17.82 °C to 18.14 °C, while the “hot region” temperature rose from 17.80 °C to 22.10 °C, resulting in a $\Delta T_{ad}$ of 3.96 °C. In Test 2, similar thermal patterns were observed with the “cold region” temperature changing from 17.38 °C to 18.02 °C and the “hot region” from 17.44 °C to 22.33 °C, resulting in a $\Delta T_{ad}$ of 4.31 °C. The consistency of these results demonstrates good repeatability of the elastocaloric response.

#### 3.1.2. Configuration 2 (δ = 33 mm): Test 3 and Test 4

**Figure 6:**
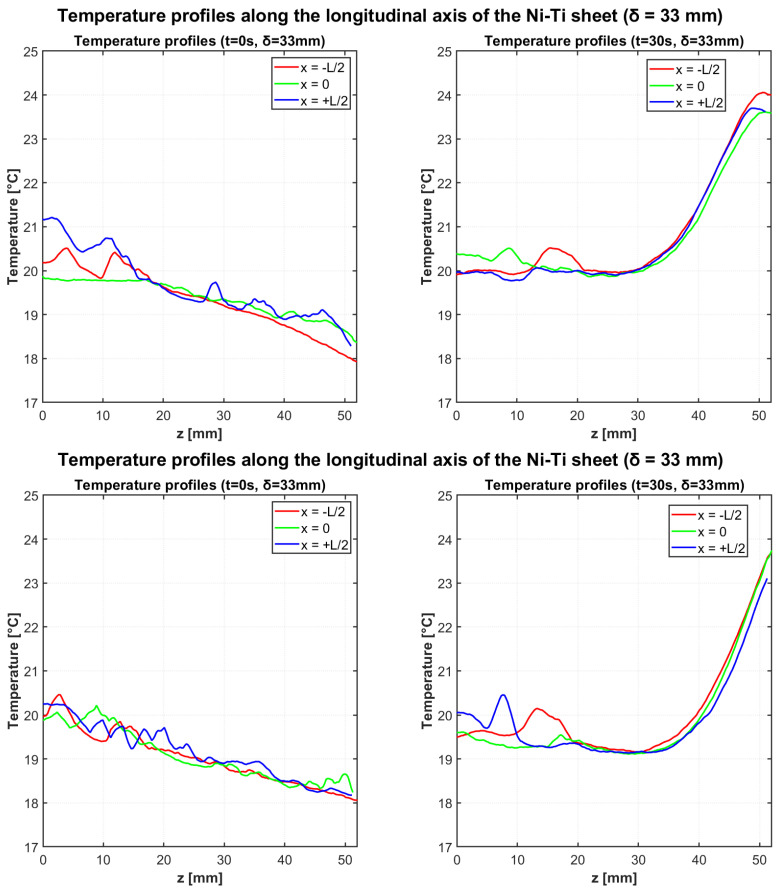
Temperature profiles along the longitudinal axis of the Ni-Ti sheet at t = 0 s (**left**) and t = 30 s (**right**) for two independent heating tests with deflection δ = 33 mm.

Figure 6 presents the temperature profiles for both heating tests with the increased deflection of δ = 33 mm. Similar to the previous configuration, the thermal gradients develop along the longitudinal axis, but with enhanced temperature variations due to the greater mechanical loading.

Table 6 and Table 7 summarize the temperature data for both tests with δ = 33 mm configuration.

Test 3 showed the “cold region” temperature varying from 18.25 °C to 19.94 °C, while the “hot region” changed from 19.95 °C to 24.00 °C, producing a $\Delta T_{ad}$ of 4.06 °C. Test 4 confirmed the consistency of the elastocaloric effect with temperature variations from 18.15 °C to 19.50 °C in the “cold region” and from 19.84 °C to 23.95 °C in the “hot region”, resulting in a $\Delta T_{ad}$ of 4.45 °C.

The adiabatic temperature change ($\Delta T_{ad}$), defined as the temperature difference between the hot and cold regions, averaged 4.14 °C for the δ = 31 mm configuration (3.96 °C and 4.31 °C in the two trials) and 4.26 °C for the δ = 33 mm configuration (4.06 °C and 4.45 °C in the two trials).

### 3.2. Cooling Cycle Analysis

The cooling cycle analysis focused on the thermal homogenization dynamics following the removal of mechanical loading. After achieving significant temperature gradients through cyclic bending, the mechanical load was removed, and the natural cooling process was monitored for 60 s to evaluate the thermal relaxation characteristics of the Ni-Ti sheet.

**Figure 7:**
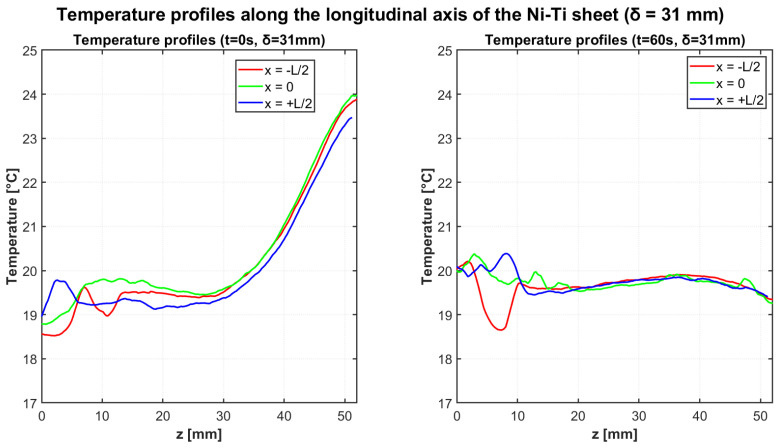
Temperature profiles along the longitudinal axis of the Ni-Ti sheet at t = 0 s (**left**) and t = 60 s (**right**) for the cooling test with deflection δ = 31 mm. Initially pronounced thermal gradients (with maximum temperatures of approximately 24 °C at the fixed end) evolve toward thermal equilibrium, with nearly uniform temperatures of about 20 °C after 60 s.

Figure 7 illustrates the temperature profiles along the three longitudinal axes at the beginning (t = 0 s) and end (t = 60 s) of the cooling cycle for the δ = 31 mm configuration. The thermal distribution at t = 0 s exhibits the characteristic gradient established during the heating cycle, with elevated temperatures near the fixed end of the cantilever. After 60 s of cooling, the profiles demonstrate substantial thermal homogenization, with the temperature gradient significantly reduced across the entire length of the specimen.

Table 8 provides quantitative data for the temperature profiles at the beginning and end of the cooling cycle for the δ = 31 mm configuration.

**Figure 8:**
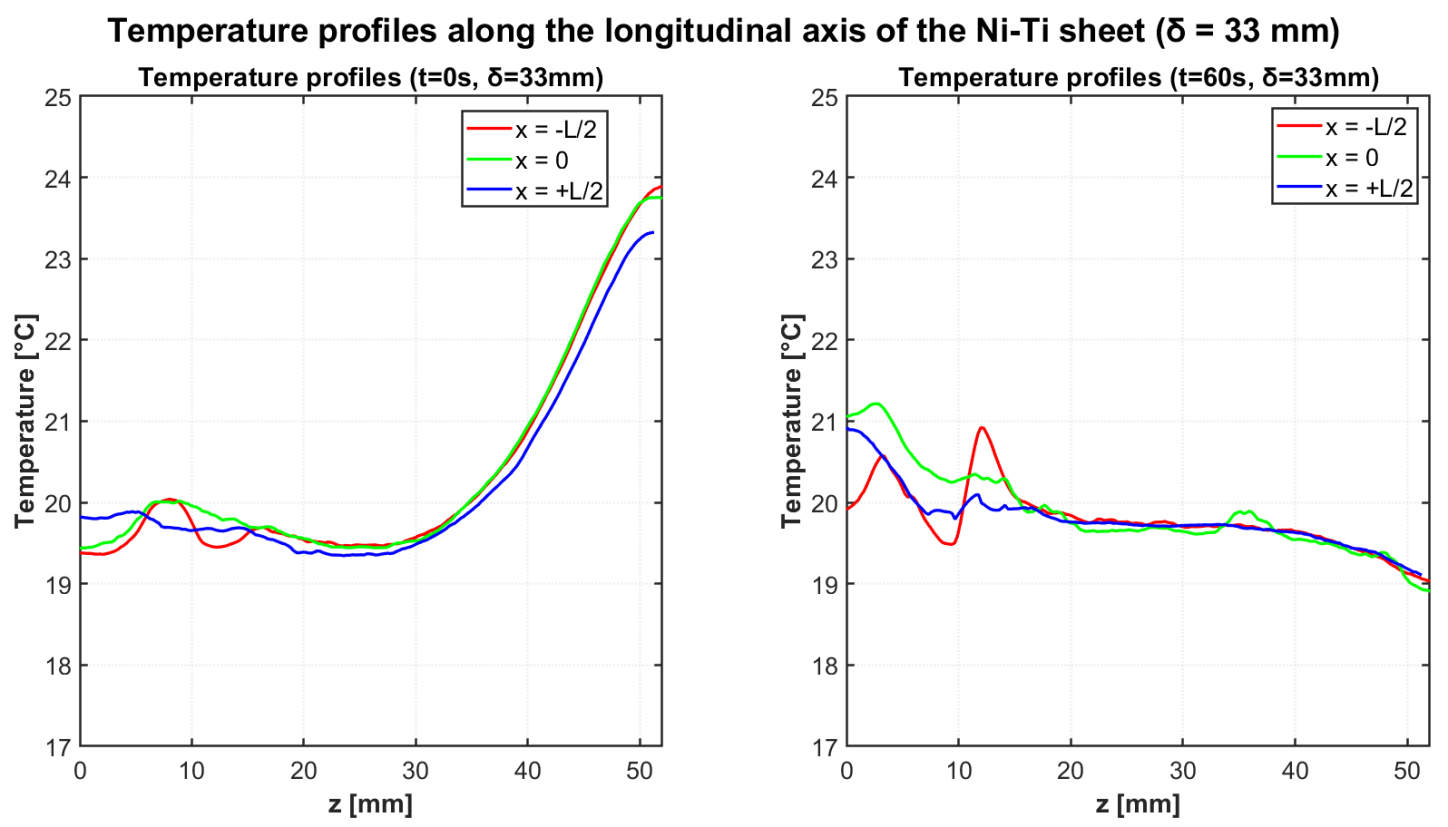
Temperature profiles along the longitudinal axis of the Ni-Ti sheet at t = 0 s (**left**) and t = 60 s (**right**) for the cooling test with deflection δ = 33 mm. The initially steeper thermal gradient (with maximum temperatures reaching approximately 24 °C) reduces substantially after 60 s of cooling, though with a slightly more pronounced residual gradient compared to the δ = 31 mm configuration.

Similar thermal evolution was observed for the δ = 33 mm configuration, as shown in Figure 8. The initial temperature gradient was slightly more pronounced compared to the δ = 31 mm configuration, consistent with the enhanced elastocaloric response observed during the heating cycle. Over the 60-s cooling period, significant thermal homogenization occurred, though a small residual gradient remained detectable.

Table 9 summarizes the temperature data for the δ = 33 mm cooling test.

To further characterize the thermal homogenization process, the specific regions of interest (“cold” and “hot”) were analyzed throughout the cooling cycle. Table 10 and Table 11 present temperature data for these regions at the beginning and end of the cooling period for both deflection configurations.

The initial temperature difference between the “hot” and “cold” regions was measured as 4.13 °C for the δ = 31 mm configuration and 4.43 °C for the δ = 33 mm configuration. These values are consistent with the adiabatic temperature variations observed during the heating cycles, confirming the reproducibility of the elastocaloric effect across different test phases.

The thermal homogenization process was highly efficient in both configurations, with the temperature gradient reducing to just 0.13 °C after 60 s for the δ = 31 mm configuration. For the δ = 33 mm configuration, a slightly larger residual gradient of 0.68 °C remained after the same cooling period, suggesting that higher initial thermal gradients may require longer homogenization times or indicate altered thermal transport characteristics at increased stress levels.

**Figure 9:**
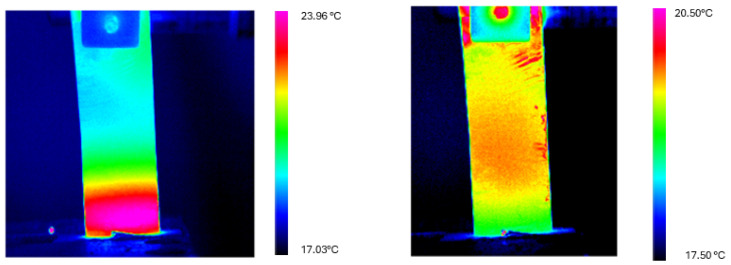
Thermograms recorded on the Ni-Ti sheet at t = 0 s (**left**) and t = 60 s (**right**) during the cooling cycle for the δ = 31 mm configuration. The color scale represents temperature in °C. The images clearly demonstrate the transition from a pronounced thermal gradient (with localized heating at the fixed end) to an almost uniform temperature distribution after 60 s of natural cooling.

**Figure 10:**
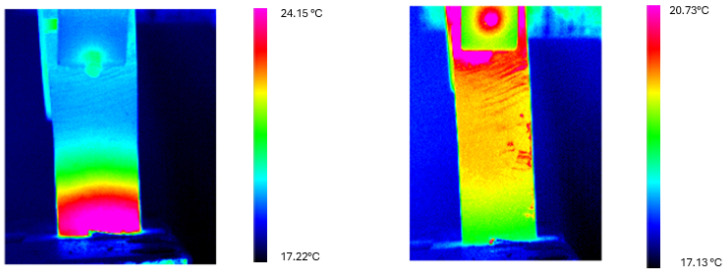
Thermograms recorded on the Ni-Ti sheet at t = 0 s (**left**) and t = 60 s (**right**) during the cooling cycle for the δ = 33 mm configuration. The color scale represents temperature in °C. While significant thermal homogenization occurs, a residual gradient remains detectable after 60 s, in contrast to the more complete equilibration observed in the δ = 31 mm configuration.

Figure 9 and Figure 10 present thermograms of the cooling process for both deflection configurations, illustrating the progressive homogenization of the temperature.

The statistical analysis of the cooling processes revealed interesting insights into the thermal transport characteristics of the Ni-Ti sheets. The ratio of standard deviations between “hot” and “cold” regions ($\gamma_{std}$) was relatively balanced at the beginning of the cooling cycle (0.87 for δ = 31 mm and 0.73 for δ = 33 mm), but increased substantially by the end of the cooling period (2.68 for δ = 31 mm and 3.43 for δ = 33 mm). This evolution suggests that the thermal homogenization process produces greater spatial temperature variation in the previously heated regions, potentially due to complex interactions between the reversing phase transformation and thermal conduction mechanisms.

The temperature ratio ($\gamma_{T}$) between “hot” and “cold” regions decreased from 1.22 to 1.01 for the δ = 31 mm configuration and from 1.23 to 1.04 for the δ = 33 mm configuration. These values demonstrate the effectiveness of thermal homogenization in both cases, with slightly more complete equilibration in the lower deflection configuration.

The cooling dynamics observed in these experiments provide valuable insights into the thermal transport characteristics of Ni-Ti sheet elements. The rapid thermal equilibration demonstrated by the Ni-Ti sheets, particularly in the δ = 31 mm configuration, suggests good thermal transport characteristics that contribute to understanding the thermomechanical behavior of these materials.

### 3.3. Evaluation of Elastocaloric Performance

To fully characterize the elastocaloric effect results obtained in the bending load configuration and assess the thermomechanical response, a comprehensive evaluation was conducted on the experimental data. This analysis focused on three key aspects: the repeatability of the thermal response, quantitative comparison between the different deflection configurations, and the assessment of energy transfer characteristics.

#### 3.3.1. Repeatability Assessment

The consistency of the elastocaloric effect was evaluated by analyzing the variation in adiabatic temperature change ($\Delta T_{ad}$) across the independent tests for each deflection configuration. Table 12 summarizes the relative deviations, which remained below 5% for both configurations, demonstrating good repeatability of the elastocaloric response.

The temperature ratio ($\gamma_{T}$) between hot and cold regions showed consistent values of 1.22–1.24 for δ = 31 mm and 1.20–1.23 for δ = 33 mm across repeated tests, confirming measurement consistency. The marginally higher relative deviation observed in the δ = 33 mm configuration (4.58% versus 4.23%) suggests that increased deflection may introduce slightly greater variability in the thermal response, potentially due to more complex transformation dynamics at higher stress levels.

#### 3.3.2. Quantitative Comparison Between Deflection Configurations

Direct comparison between the two deflection configurations revealed systematic differences in thermal response characteristics. The higher deflection (δ = 33 mm) produced greater adiabatic temperature variations, with an average increase of 0.12 °C (approximately 2.9%) compared to the δ = 31 mm configuration. This enhancement, though modest in absolute terms, demonstrates positive correlation between deflection magnitude and elastocaloric response intensity.

The thermal homogeneity, quantified through the ratio of standard deviations between hot and cold regions ($\gamma_{std}$), showed notable differences between configurations. During the heating phase, the $\gamma_{std}$ values ranged from 3.0–3.5 for the δ = 31 mm configuration and 4.2–7.0 for the δ = 33 mm configuration. This increase in thermal heterogeneity at higher deflection levels reflects the more complex stress distribution and transformation patterns occurring under greater mechanical loading.

During the cooling phase, both configurations demonstrated efficient thermal homogenization, though the δ = 33 mm configuration retained a slightly larger residual gradient (0.68 °C vs. 0.13 °C after 60 s), indicating that higher deflection magnitudes influence both the intensity and the subsequent thermal transport dynamics.

#### 3.3.3. Energy Transfer Quantification

The thermodynamic characteristics of the elastocaloric effect were evaluated through calculation of the heat generated during the martensitic transformation using the methodology previously described in Section 2.5.

The heat exchange was estimated for the portion of the specimen experiencing significant temperature change (approximately 6 mm from the fixed end of the cantilever).

Table 13 presents the calculated heat values for all tests conducted in this study.

The heat generated varied from 1.48 J to 1.66 J, depending on the configuration and specific test conditions. These values, while modest in absolute terms, demonstrate significant energy density when normalized by the active material volume. For the transforming region, this corresponds to energy densities of 12.3–13.8 J/cm^3^, providing quantitative characterization of the thermomechanical coupling efficiency in the bending configuration.

The analysis revealed a consistent correlation between deflection magnitude and heat generation, with the δ = 33 mm configuration producing approximately 7–12% more heat than the δ = 31 mm configuration under equivalent test conditions. This relationship demonstrates the tunability of the elastocaloric response through mechanical loading parameters.

## 4. Discussion

The experimental investigation conducted in this research has provided comprehensive insights into the elastocaloric behavior of Ni-Ti sheets under cyclic bending loads. This section interprets these findings within the broader context of elastocaloric phenomena, analyzing the thermal characteristics and implications of the observed thermal responses.

### 4.1. Elastocaloric Effect Characterization in Bending Configuration

The experimental results obtained from the cyclic bending configuration demonstrate significant elastocaloric effects in Ni-Ti sheets, with adiabatic temperature variations ($\Delta T_{ad}$) of 4.14 °C and 4.26 °C for deflections of 31 mm and 33 mm, respectively. These temperature variations are consistent with the stress-induced martensitic transformation occurring during cyclic loading, which is characteristic of elastocaloric behavior in SMA [3].

Unlike conventional uniaxial tension or compression configurations commonly reported in literature [11], the bending loading configuration employed in this study generates a non-uniform stress distribution across the Ni-Ti sheet cross-section. This gradient ranges from tensile stress at the outer fibers to compressive stress at the inner fibers, with a neutral axis experiencing minimal stress. The thermogram revealed that despite this heterogeneous stress field, the elastocaloric response maintains good reproducibility with relative deviations below 5%.

The thermal gradients observed along the longitudinal axis of the Ni-Ti sheets align with the expected stress distribution in a cantilever beam subjected to bending loads, with maximum temperatures consistently occurring near the fixed end where stress is highest. This pattern confirms that the stress-induced phase transformation is the primary source of the observed thermal effects, rather than frictional heating. The temperature profiles exhibited remarkable consistency across the transverse direction (x = −L/2, x = 0, x = L/2), suggesting that the elastocaloric effect in the bending configuration propagates uniformly across the width of the Ni-Ti sheet. This homogeneity in the transverse direction represents an advantageous characteristic for thermal transport analysis, as it maximizes the effective surface area available for thermal transfer.

### 4.2. Thermodynamic Aspects and Literature Comparison

The adiabatic temperature variations ($\Delta T_{ad}$) measured in our study (4.14–4.26 °C) can be compared with the limited literature available on elastocaloric effects in Ni-Ti alloys under bending configurations.

Sharar et al. [12] demonstrated temperature reductions up to 11.3 °C in Ni-Ti wire under four-point bending, although their experimental setup utilized a different specimen geometry (wire versus sheet).

Direct comparison with other bending studies reveals limited availability of comparable data. Cheng et al. [19] characterized the elastocaloric effect of Ni_50.8_Ti_49.2_ (at.%) alloy sheets with a thickness of 0.5 mm under bending in a custom-built rotating bending device and recorded 8–10 K of temperature difference between the highest temperature on the hot area and the lowest temperature on the cool area on the continuously rotating Ni-Ti sheets. However, this temperature difference represents the spatial gradient across the sheet rather than the adiabatic temperature change at a specific location, making direct comparison with our $\Delta T_{ad}$ values challenging.

Our temperature changes are substantially lower than those reported for uniaxial tensile loading [19], where maximum adiabatic temperature change values of 31 K and 23 K were recorded in Brayton-like cyclic loadings under maximum applied stresses of 600 and 400 MPa, respectively. This comparison highlights the inherent trade-off between the reduced actuation force achieved in bending configurations and the magnitude of the elastocaloric temperature change.

The thermal homogenization dynamics observed in our cooling tests provide unique insights not commonly reported in the elastocaloric literature. Our finding that the temperature gradient reduced from 4.13 °C to just 0.13 °C over 60 s for the 31 mm deflection configuration represents a quantitative characterization of thermal equilibration that is rarely documented in detail. The slightly slower equilibration observed in the 33 mm configuration (residual gradient of 0.68 °C after 60 s) suggests that higher initial thermal gradients may alter the thermal transport properties, though comparable data from other bending studies is not available for direct comparison.

The energy density values calculated from our experimental results (12.3–13.8 J/cm^3^) provide a basis for thermodynamic comparison, though specific energy density data for bending configurations is scarce in the literature. Li et al. [14], utilizing a coil-bending approach, achieved continuous cold outlet air with a temperature drop of 10.6 K and a specific cooling power of 2.5 W g^−1^ at a low specific driving force of 26 N g^−1^, but did not report energy density values for direct comparison.

A notable finding of our study is the non-linear relationship between mechanical input and thermal response, with a 6.5% increase in deflection magnitude resulting in a 7–12% increase in heat generation. This non-linear behavior appears to be a characteristic feature of elastocaloric materials under various loading modes, though specific data on this relationship for bending configurations is not readily available in the literature.

The influence of material dimensions on cooling performance is another important parameter highlighted in recent literature. Cheng et al. [19] systematically investigated the effect of Ni-Ti sheet thickness (0.2–0.5 mm) on both the elastocaloric response and the resulting cooling performance in a rotating bending system. Their study demonstrated that sheet thickness significantly affects both the adiabatic temperature change and the fatigue life, with thinner sheets exhibiting smaller temperature variations but potentially improved cycling stability. This dimensional effect aligns with our observations on the influence of geometric parameters on thermal response and suggests potential optimization pathways for future elastocaloric cooling systems based on bending actuation.

## 5. Conclusions

This study presents a systematic thermographic characterization of elastocaloric behavior in Ni-Ti sheet elements subjected to cyclic bending loading. The experimental investigation employed passive infrared thermography to document and quantify thermal response characteristics under controlled mechanical deflection conditions.

**Quantitative thermal characterization:** The experimental investigation demonstrated reproducible elastocaloric responses with consistent adiabatic temperature variations of 4.14 °C for deflection δ = 31 mm and 4.26 °C for deflection δ = 33 mm, achieving relative deviations below 5%. These temperature changes, while modest compared to uniaxial loading configurations reported in the literature, provide quantitative baseline data for elastocaloric performance assessment in bending-loaded configurations.

**Thermal homogenization dynamics:** The investigation revealed efficient thermal transport characteristics, with temperature gradients reducing from 4.13 °C to 0.13 °C (δ = 31 mm) and from 4.43 °C to 0.68 °C (δ = 33 mm) over a 60 s cooling period. This rapid thermal equilibration demonstrates the material’s capacity for thermal redistribution following mechanical loading cessation.

**Energy transfer quantification:** Thermodynamic analysis provided heat generation values ranging from 1.48 J to 1.66 J across all test configurations, corresponding to energy densities of 12.3–13.8 J/cm^3^ within the active transformation region. A systematic correlation was documented between deflection magnitude and heat generation, with the δ = 33 mm configuration producing 7–12% more heat than the δ = 31 mm configuration.

**Spatiotemporal thermal mapping:** The comprehensive thermal analysis methodology provided detailed documentation of thermal evolution patterns across the entire Ni-Ti sheet surface, revealing localized transformation patterns and thermal distribution gradients. The observed thermal distributions aligned with expected stress patterns in cantilever beam configurations, confirming that stress-induced martensitic transformation represents the primary source of thermal effects.

**Methodological contributions:** The spatiotemporal thermographic approach developed in this research provides a foundation for quantitative assessment of elastocaloric performance in complex loading scenarios. Unlike previous investigations that primarily focused on system-level performance metrics, this study offers detailed thermal characterization methodology for bending-loaded shape memory alloy elements.

The documented thermal responses establish experimental data that may support future investigations in elastocaloric cooling applications utilizing bending actuation configurations. The findings contribute quantitative thermal characterization data for elastocaloric behavior in Ni-Ti sheet elements under cyclic bending conditions.

## Figures and Tables

**Table 1:** Thermographic acquisition parameters.

Parameter	Value
Camera-specimen distance [mm]	200
Background temperature [°C]	25
Pixel size [μm]	13
Detector wavelength range [μm]	2.60–5.10
Housing temperature [°C]	28–32
Sampling frequency [Hz]	25

**Table 2:** Operational parameters for the experimental setup.

Parameter	Configuration 1	Configuration 2
Free specimen length (h) [mm]	52	52
Deflection (δ) [mm]	31	33
Rotational speed [rpm]	1800	1800
Thermographic camera distance [mm]	200	200
Sampling frequency [Hz]	25	25
Heating cycle duration [s]	30	30
Cooling cycle duration [s]	60	60

**Table 3:** Adiabatic temperature variations from heating tests.

Configuration	Test	Δ*T_ad_* [°C]	Mean Δ*T_ad_* [°C]
*δ* = 31 mm	Test 1	3.96	4.14
	Test 2	4.31	
*δ* = 33 mm	Test 3	4.06	4.26
	Test 4	4.45	

**Table 4:** Temperature measurements at t = 0 s for both tests with δ = 31 mm configuration.

Test	Profile	T min [°C]	T max [°C]	Mean [°C]	St. Dev [°C]
Test 1	x = −L/2	17.07	18.06	17.53	0.22
	x = 0	17.13	18.17	17.61	0.24
	x = L/2	17.14	17.87	17.60	0.23
Test 2	x = −L/2	17.32	17.74	17.50	0.09
	x = 0	17.32	17.82	17.52	0.10
	x = L/2	17.34	17.58	17.44	0.09

**Table 5:** Temperature measurements at t = 30 s for both tests with δ = 31 mm configuration.

Test	Profile	T min [°C]	T max [°C]	Mean [°C]	St. Dev [°C]
Test 1	x = −L/2	18.01	22.61	19.36	1.50
	x = 0	17.71	22.87	19.60	1.57
	x = L/2	18.53	22.80	19.75	1.42
Test 2	x = −L/2	17.98	22.54	19.57	1.51
	x = 0	17.75	23.02	19.57	1.67
	x = L/2	18.35	22.73	19.62	1.49

**Table 6:** Temperature measurements at t = 0 s for both tests with δ = 33 mm configuration.

Test	Profile	T min [°C]	T max [°C]	Mean [°C]	St. Dev [°C]
Test 3	x = −L/2	18.06	20.06	19.24	0.56
	x = 0	17.17	21.10	19.28	0.78
	x = L/2	17.51	20.33	19.53	0.73
Test 4	x = −L/2	18.02	20.33	18.98	0.64
	x = 0	17.84	21.16	19.02	0.73
	x = L/2	18.02	20.33	18.98	0.64

**Table 7:** Temperature measurements at t = 30 s for both tests with δ = 33 mm configuration.

Test	Profile	T min [°C]	T max [°C]	Mean [°C]	St. Dev [°C]
Test 3	x = −L/2	18.66	23.46	20.61	1.26
	x = 0	19.85	24.08	20.68	1.39
	x = L/2	19.76	23.89	20.91	1.37
Test 4	x = −L/2	18.84	23.00	20.83	1.22
	x = 0	19.03	24.18	20.16	1.39
	x = L/2	18.84	23.84	20.00	1.03

**Table 8:** Temperature data along the three longitudinal profiles (x = −L/2, x = 0, x = L/2) for the δ = 31 mm cooling test at t = 0 s and t = 60 s.

Time	Profile	T min [°C]	T max [°C]	Mean [°C]	St. Dev [°C]
t = 0 s	x = −L/2	19.12	23.86	20.16	1.30
	x = 0	18.54	23.65	20.18	1.37
	x = L/2	19.38	23.93	20.55	1.31
t = 60 s	x = −L/2	19.23	20.50	19.70	0.28
	x = 0	18.59	20.23	19.59	0.31
	x = L/2	19.10	20.41	19.70	0.27

**Table 9:** Temperature data along the three longitudinal profiles (x = −L/2, x = 0, x = L/2) for the δ = 33 mm cooling test at t = 0 s and t = 60 s.

Time	Profile	T min [°C]	T max [°C]	Mean [°C]	St. Dev [°C]
t = 0 s	x = −L/2	19.13	23.87	20.22	1.27
	x = 0	19.12	24.50	20.39	1.50
	x = L/2	19.10	24.31	20.25	1.46
t = 60 s	x = −L/2	19.04	20.98	19.83	0.41
	x = 0	19.03	21.02	19.80	0.39
	x = L/2	18.90	21.13	19.90	0.39

**Table 10:** Temperature data in the “cold” and “hot” regions for the δ = 31 mm cooling test at t = 0 s and t = 60 s.

Region	Time	Mean [°C]	St. Dev [°C]	Area [mm^2^]
Cold region	t = 0 s	18.99	0.67	182.60
	t = 60 s	19.14	0.25	182.60
Hot region	t = 0 s	23.12	0.58	182.60
	t = 60 s	19.27	0.67	182.60

**Table 11:** Temperature data in the “cold” and “hot” regions for the δ = 33 mm cooling test at t = 0 s and t = 60 s.

Region	Time	Mean [°C]	St. Dev [°C]	Area [mm^2^]
Cold region	t = 0 s	19.27	1.02	182.60
	t = 60 s	19.21	0.23	182.60
Hot region	t = 0 s	23.70	0.74	182.60
	t = 60 s	19.92	0.79	182.60

**Table 12:** Analysis of the adiabatic temperature change ($\Delta T_{ad}$) for both deflection configurations.

Configuration	Mean Δ*T_ad_* [°C]	Relative Deviation [%]
δ = 31 mm	4.14	4.23
δ = 33 mm	4.26	4.58

**Table 13:** Heat generation during elastocaloric effect for all test configurations.

Test Type	Configuration	Δ*T_ad_* [°C]	Heat Generated [J]	Test Duration [s]
Heating	δ = 31 mm	3.96	1.48	30
		4.31	1.61	30
	δ = 33 mm	4.06	1.52	30
		4.45	1.66	30
Cooling	δ = 31 mm	4.13	1.54	60
	δ = 33 mm	4.43	1.65	60

## Data Availability

The original contributions presented in this study are included in the article. Further inquiries can be directed to the corresponding author.

## Appendix A. Calculation of Specific Heat Capacity for Ni-Ti Alloy

The specific heat capacity of the Ni-Ti alloy was calculated using the temperature-dependent equation reported by Smith et al. [17] for the stoichiometric NiTi phase:

(A1) $$C_{p}\left(\mathrm{NiTi}\right)=24.82+2.13\times 10^{-3}T-110010T^{-2}\;\left(J/\mathrm{g-atom}\cdot K\right)$$

This approach was necessary because the commercial supplier (Nexmetal Corporation, Sheridan, WY, USA) was unable to provide exact chemical composition details, citing proprietary manufacturing specifications. The Ni-Ti sheets employed in this investigation are the same material used in our previous study [15], where comprehensive XRD analysis was performed.

The equation (Equation (6) in Smith et al. [17]) was derived from experimental measurements using a Perkin-Elmer DSC-2 differential scanning calorimeter (Waltham, MA, USA) on high-purity Ni-Ti alloys in the temperature range 120–800 K. The equation is valid both below and above the martensitic transition range (320–370 K), excluding the anomalous heat capacity behavior during the transition itself.

At experimental temperature (T = 292.15 K, corresponding to 19 °C), Equation (A1) results in the following:

(A2) $$C_{p}=24.82+2.13\times 10^{-3}\times 292.15-110010\times {\left(292.15\right)}^{-2}=24.81\;J/\mathrm{g-atom}\cdot K$$

To convert from J/g-atom·K to J/g·K, the value was divided by the molar mass of the alloy. The compositional analysis was based on our previous comprehensive XRD characterization [15], which confirmed

- B2 austenitic phase structure with lattice parameter 3.001 ± 0.003 Å;
- Transformation temperatures consistent with near-equiatomic composition;
- Austenite finish temperature (A*_f_*) of 5 °C, characteristic of Ni-Ti alloys with approximately 50.8–51.0 at.% Ni content.

Given this characterization, three representative compositions spanning the likely compositional range were considered.

**Table A1:** Specific heat capacity calculations for different Ni-Ti compositions at 19 °C using Smith et al. [17] equation, based on compositional range determined from XRD analysis [15].

Composition	Molar Mass [g/mol]	*C_p_* [J/g·K]
NiTi (50.0–50.0 at.%)	54.74	0.4533
Ni-rich (50.8–49.2 at.%)	54.82	0.4525
Ni-rich (51.0–49.0 at.%)	54.86	0.4523

Based on the XRD-determined near-equiatomic composition with slight Ni-richness (50.8–51.0 at.% Ni), a representative value of 0.46 J/g·K was selected for the thermodynamic calculations. This value is consistent with the calculated range and represents the specific material used in both studies.
